# Extraction of critical metals from chrysotile asbestos tailings *via* an ammonium sulfate roasting-water leaching process

**DOI:** 10.1039/d6ra09542a

**Published:** 2026-11-12

**Authors:** Hongxiang Gan, Xiangyang Zhou, Fanyun Su

**Affiliations:** a School of Metallurgy and Environment, Engineering Research Center of the Ministry of Education for Advanced Battery Materials, Central South University Changsha 410083 China 1453636044@qq.com +8613278881878

## Abstract

Chrysotile asbestos tailings (CATs) are hazardous industrial wastes that contain substantial amounts of valuable critical metals, including Mg, Fe, and Ni. This study investigates the co-extraction of these metals from CATs using an ammonium sulfate ((NH_4_)_2_SO_4_) roasting-water leaching process. Key process parameters, including (NH_4_)_2_SO_4_ dosage, roasting temperature, and roasting time, were systematically optimized to maximize the leaching efficiencies of Mg, Fe, and Ni. The phase transformations and microstructural evolution during roasting were characterized by X-ray diffraction (XRD), scanning electron microscopy (SEM), and thermogravimetric analysis (TG). Under the optimal conditions, the leaching efficiencies of Mg, Fe, and Ni all exceeded 93%. XRD and TG analyses revealed that the roasting process effectively decomposed the chrysotile structure and transformed the metal-bearing phases into water-soluble Fe_2_(SO_4_)_3_, NiSO_4_, and magnesium sulfate, which were subsequently dissolved during water leaching. The results demonstrate that the proposed process achieves high simultaneous extraction efficiencies for multiple valuable metals while minimizing secondary pollution. This study provides a feasible and cleaner production route for the sustainable utilization of CATs.

## Abstract 2

A low-temperature (NH_4_)_2_SO_4_ roasting process was developed for CATs. At 550 °C, the fibrous structure was destroyed, achieving efficient co-extraction of Mg, Fe, and Ni while rejecting Si.

## Graphical abstract

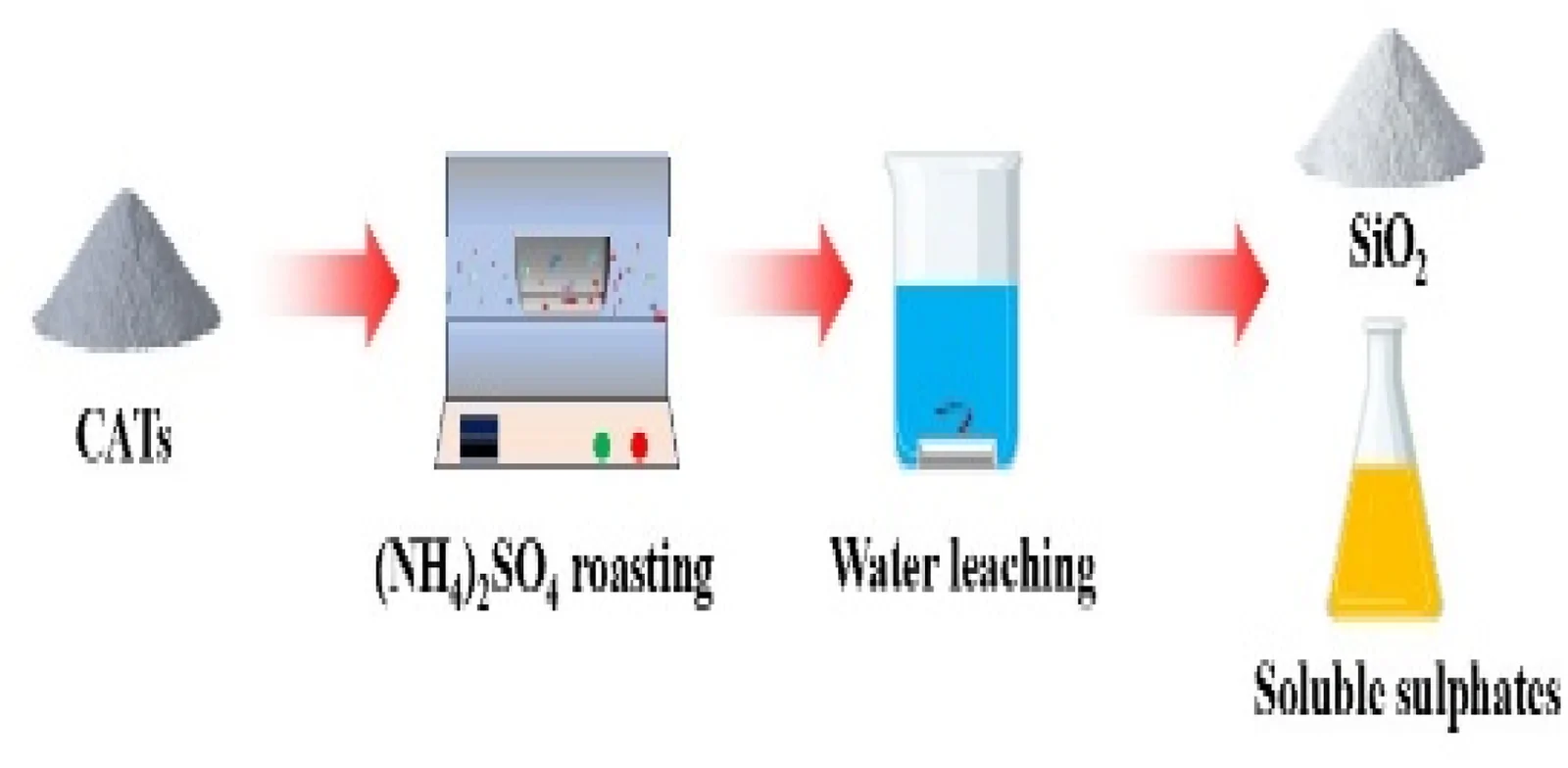

## 1. Introduction

Chrysotile asbestos tailings (CATs) are a typical industrial solid waste generated during asbestos mining and processing. It is estimated that millions of tons of these tailings have been accumulated worldwide, occupying vast land areas and posing significant environmental risks due to their biotoxicity.^1–4^ Meanwhile, CATs contain substantial amounts of valuable metallic elements, particularly Mg, Fe, and Ni, making them an underutilized secondary resource.^5,6^ The main mineral phase in these tailings is chrysotile (Mg_3_Si_2_O_5_(OH)_4_), a magnesium silicate mineral that also incorporates Fe and Ni in its lattice structure.^7^ Therefore, the sustainable disposal and resource utilization of CATs have become an urgent issue from both environmental and economic perspectives.

Various methods have been developed for the treatment and resource utilization of CATs. Direct landfilling or stockpiling remains the most common practice in many mining areas, but this approach occupies large land areas and poses long-term environmental risks.^8^ For metal extraction, acid leaching using hydrochloric acid or sulfuric acid has been widely studied. However, this route suffers from severe equipment corrosion, high acid consumption, and the generation of large volumes of acidic wastewater.^9–11^ Alkaline roasting-water leaching processes have also been reported, but they typically require higher temperatures and consume large amounts of alkali reagents, resulting in increased costs.^3,12^ Pyrometallurgical approaches such as smelting or vitrification can effectively immobilize hazardous components, yet they are energy-intensive and often fail to recover valuable metals efficiently.^13,14^ Flotation and gravity separation have been employed for physical beneficiation of CATs, but their separation efficiency is limited due to the fine particle size and complex mineral associations.^5^ In addition, carbonation methods have been explored for CO_2_ sequestration using CATs as a feedstock, though metal recovery is not the primary objective of these processes.^15,16^ In contrast, the ammonium sulfate ((NH_4_)_2_SO_4_) roasting-water leaching process has emerged as a promising alternative for metal recovery from CATs. This method offers several advantages, including relatively low roasting temperature (typically 400–650 °C), high metal extraction efficiency, good reaction selectivity, environmental friendliness, and low energy consumption. During roasting, (NH_4_)_2_SO_4_ reacts with metal-bearing minerals in CATs to form water-soluble sulfates, which are subsequently recovered by water leaching. The ammonia and sulfur oxides generated during roasting can be recycled, further enhancing the economic and environmental viability of the process.

Recent studies have investigated the application of (NH_4_)_2_SO_4_ roasting for metal extraction from various minerals and industrial wastes. For laterite nickel ores, the process achieved leaching rates of 90.8% for Ni, 85.4% for Co, and 86.7% for Mn, while Fe leaching was intentionally suppressed to only 9.98%.^17,18^ In a systematic review, the (NH_4_)_2_SO_4_ roasting method has been recognized as a clean and efficient technique for recovering valuable metals from low-grade mineral resources and tailings.^19^ More recently, Chu *et al.* reported a selective extraction process for Mg^2+^ from CATs, achieving an Mg leaching rate of 84.18% at 650 °C while effectively inhibiting the co-dissolution of impurity ions, with Fe leaching rates as low as 0.04%.^2^ Furthermore, NH_4_HSO_4_ roasting-water leaching has been shown to simultaneously extract Ni, Cu and Co from nickel concentrate by converting them into water-soluble metal–ammonium complexes.^20^ On this basis, (NH_4_)_2_SO_4_ was preferentially selected for serpentine roasting in this work because it decomposes at moderate temperatures to release active sulfate species (SO_3_/SO_2_ and NH_4_HSO_4_/(NH_4_)_2_S_2_O_7_) that can selectively sulfatize Mg while leaving silica and Fe largely insoluble. It is also a solid, easy-to-handle reagent that is less corrosive than concentrated H_2_SO_4_ and avoids the chloride-related corrosion associated with NH_4_Cl. However, most existing studies have focused on the selective extraction of magnesium while suppressing the leaching of iron and other metals. The simultaneous co-extraction of multiple valuable metals (Mg, Fe, and Ni) from CATs *via* (NH_4_)_2_SO_4_ roasting has received comparatively less attention.

In this study, a systematic investigation was conducted on the co-extraction of Mg, Fe, and Ni from CATs using (NH_4_)_2_SO_4_ roasting followed by water leaching. Key process parameters, including (NH_4_)_2_SO_4_ dosage, roasting temperature, and roasting time, were optimized to maximize the leaching rates of these valuable metals. The phase transformation and microstructural evolution during roasting were characterized by X-ray diffraction (XRD), scanning electron microscopy (SEM), and thermogravimetric analysis (TG). Under the optimal conditions at 550 °C, the leaching rates of Mg, Fe, and Ni all exceeded 93%. XRD and TG analyses revealed that the roasting process effectively decomposed the chrysotile structure and transformed the metal-bearing phases into water-soluble Fe_2_(SO_4_)_3_, NiSO_4_, and magnesium sulfate, which were subsequently dissolved during water leaching. The results demonstrate that the proposed process not only achieves high co-extraction efficiencies for Mg, Fe, and Ni but also offers a promising route for the environmentally sustainable utilization of CATs.

## 2. Experimental section

### 2.1 Materials and analysis

The CATs used in this study originated from a mining operation in Mangya City, Qinghai Province, China. The chemical composition of the raw CATs was determined by inductively coupled plasma optical emission spectrometry (ICP-OES) following acid digestion, and the results are presented in Table 1. The morphology of the pretreated CATs was examined by scanning electron microscopy (SEM, as shown in Fig. 1), which revealed irregularly shaped particles with rough surfaces and a size distribution predominantly in the range of 20–50 µm, consistent with the sieving results. The mineralogical phases of the raw CATs were identified by X-ray diffraction (XRD) analysis (Fig. 3(a)), confirming that chrysotile (Mg_3_Si_2_O_5_(OH)_4_) was the dominant mineral phase. Prior to the experiments, the as-received CATs were subjected to a series of pretreatment steps, including jaw crushing, ball milling, and sieving, to obtain a homogeneous powder with a particle size of less than 74 µm (passing 200 mesh). (NH_4_)_2_SO_4_ employed in the roasting experiments was of analytical grade and purchased from Sinopharm Chemical Reagent Co., Ltd, and was used as received without further purification. Deionized water with a resistivity of 18.2 MΩ cm, produced by a Milli-Q water purification system, was used throughout the experiments for all water leaching operations and solution preparations. To ensure the reliability and reproducibility of the experimental results, all leaching experiments were performed in triplicate, and the average values along with standard deviations were reported.

**Table 1:** Elemental distribution of CATs

Element	Mg	Si	Fe	Ni
Content (wt%)	23.3	17.4	2.8	0.2

**Fig. 1:**
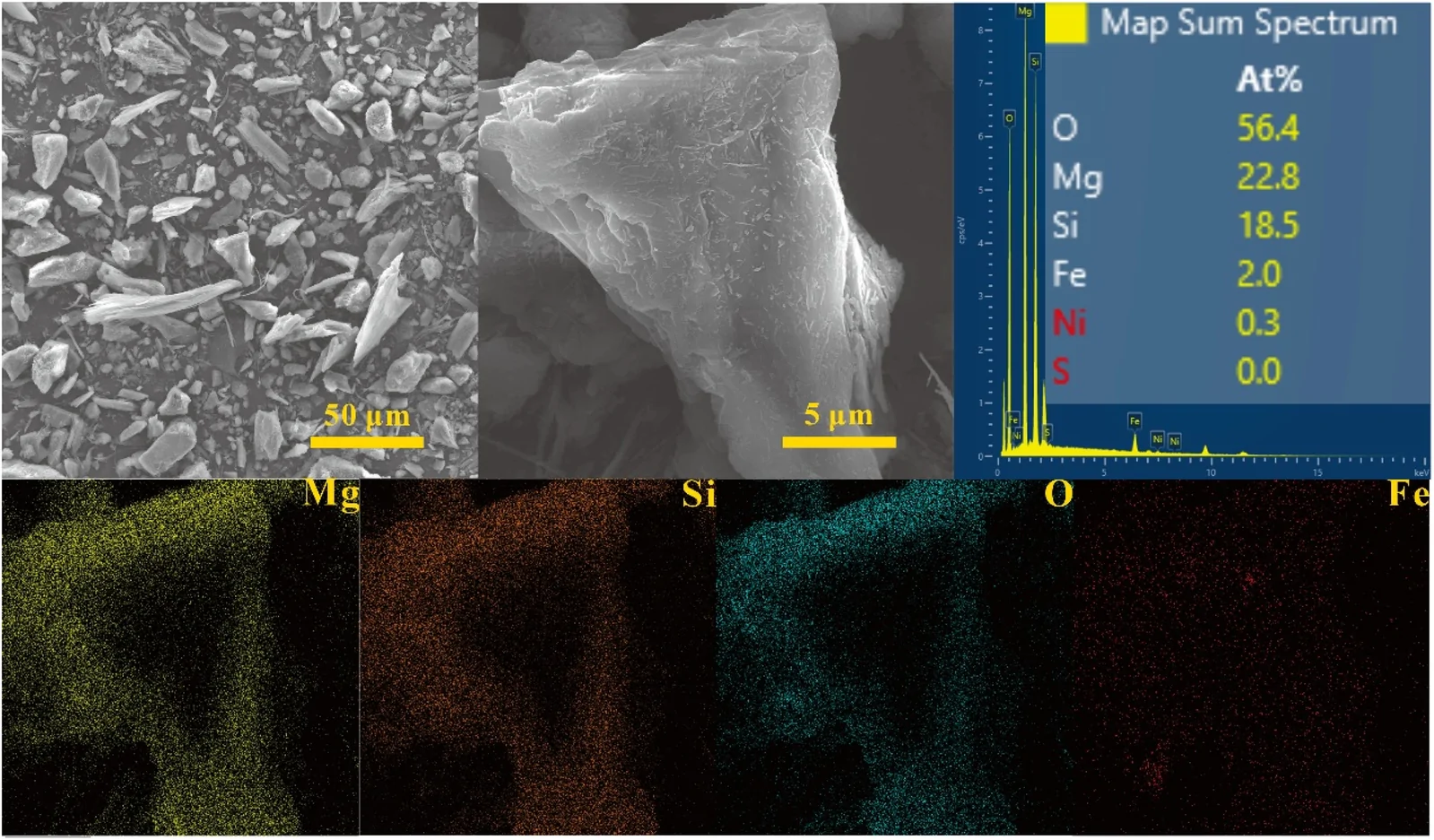
SEM images of CATs powder.

### 2.2 Experimental procedure

The roasting experiments were conducted in a muffle furnace. Raw CATs were mixed with (NH_4_)_2_SO_4_ at molar ratios of 0.5 : 1 to 2.5 : 1, then roasted at 350–750 °C for 30–150 min. The roasted products were leached with deionized water at a liquid-to-solid ratio of 10 mL g^−1^ at 25 °C for 60 min under stirring. After filtration, the concentrations of Mg, Fe, and Ni in the leachate were determined by ICP-OES, and the leaching rates were calculated accordingly. The phase composition, thermal behavior, and morphology of the samples were characterized by XRD, TG, and SEM-EDS, respectively. The effects of (NH_4_)_2_SO_4_ dosage, roasting temperature, and roasting time were optimized sequentially, and the optimal conditions were determined based on the maximum leaching rates of all three metals. In the water leaching procedure, the leaching efficiencies of valuable metals *η*_i_ was calculated by the expression below:1
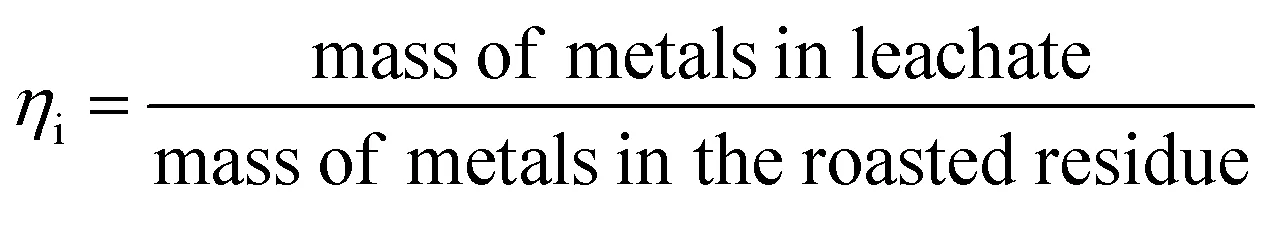


### 2.3 Analysis and characterization

The phase compositions of the CATs, roasted products, and leaching residues were determined by X-ray diffraction (XRD, Rigaku Smart Lab) with Cu Kα radiation, to clarify the phase transformation during roasting and water leaching. Thermogravimetric analysis (TG, NETZSCH STA 449F3) was performed from room temperature to 800 °C under air flow to investigate the thermal decomposition behavior and reaction temperature range of the mixture. The morphology and microstructural evolution were observed by scanning electron microscopy coupled with energy-dispersive X-ray spectroscopy (SEM-EDS, ZEISS Sigma 300), aiming to reveal the changes in particle surface and elemental distribution. The concentrations of Mg, Fe, and Ni in the leachate were measured by inductively coupled plasma optical emission spectrometry (ICP-OES, PerkinElmer Optima 8000) to calculate the leaching efficiencies. All measurements were carried out under standard operating conditions.

## 3. Results and discussion

### 3.1 Thermodynamic feasibility of (NH_4_)_2_SO_4_ roasting

The Gibbs free energy changes (Δ*G*) for the reactions between (NH_4_)_2_SO_4_ and the main metal-bearing phases in CATs, including Mg_3_Si_2_O_5_(OH)_4_, Fe_3_O_4_, Fe_2_O_3_, FeO, and NiO, were calculated as a function of temperature from 0 to 1000 °C using HSC 6.0 Chemistry software. The corresponding reaction equations are presented below. The results in Fig. 2 show that the Δ*G* values for all five reactions become negative once the temperature exceeds approximately 400 °C. Within the roasting temperature range investigated in this study, these reactions are thermodynamically favorable and possess a strong spontaneous tendency, confirming the feasibility of (NH_4_)_2_SO_4_ roasting for the extraction of Mg, Fe, and Ni from CATs. These negative Δ*G* values also provide a theoretical basis for the selection of the roasting temperature.

2 Mg_3_Si_2_O_5_(OH)_4_ + 3(NH_4_)_2_SO_4_ = 3MgSO_4_ + 2SiO_2_ + 6NH_3_(g) + 5H_2_O

3 Fe_3_O_4_ + 4(NH_4_)_2_SO_4_ = Fe_2_(SO_4_)_3_ + FeSO_4_ + 4H_2_O + 8NH_3_(g)

4 Fe_2_O_3_ + 3(NH_4_)_2_SO_4_ = Fe_2_(SO_4_)_3_ + 3H_2_O + 6NH_3_(g)

5 FeO + (NH_4_)_2_SO_4_ = FeSO_4_ + H_2_O + 2NH_3_(g)

6 NiO + (NH_4_)_2_SO_4_ = NiSO_4_ + H_2_O + 2NH_3_(g)

**Fig. 2:**
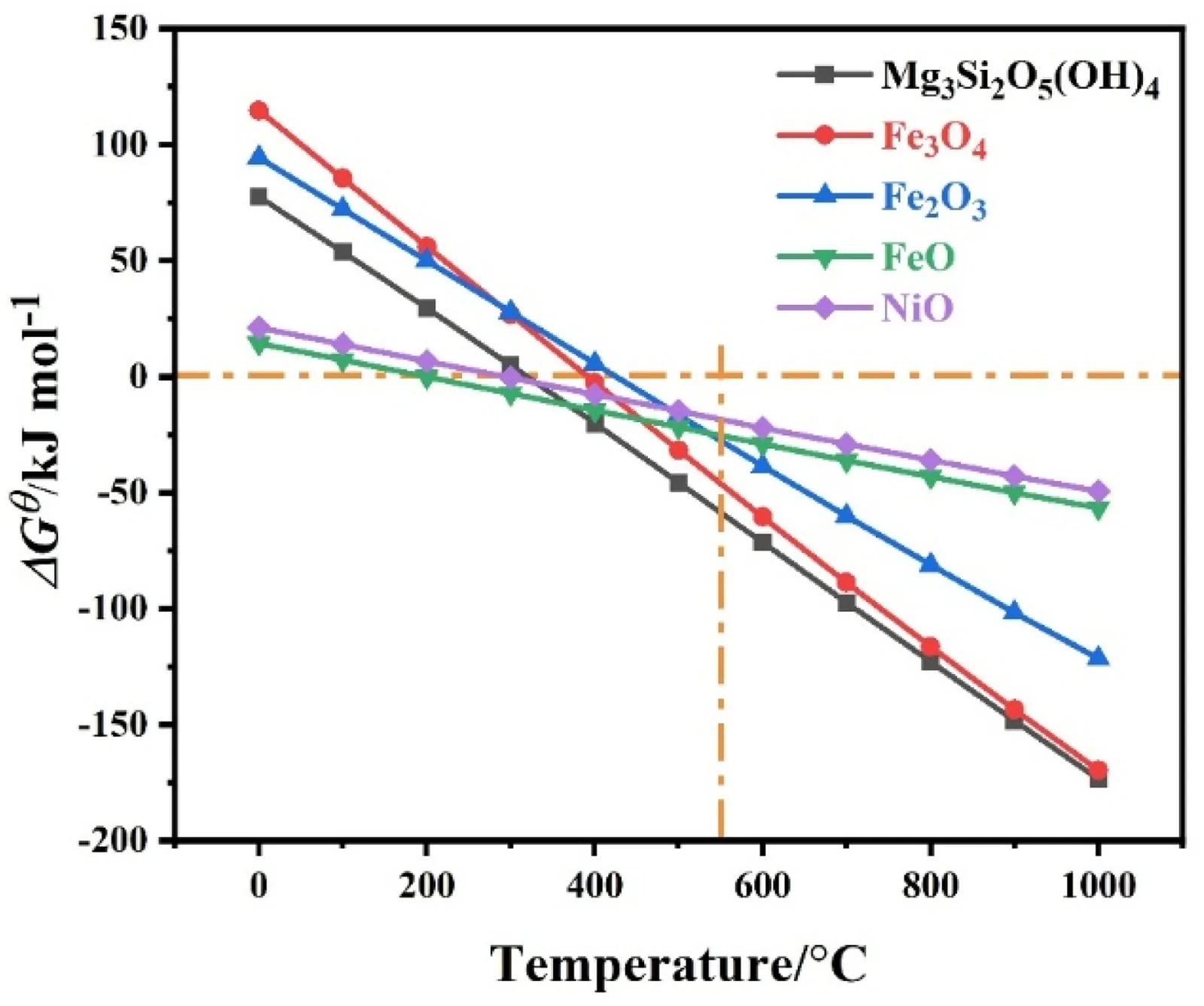
Thermodynamic data for the reactions of various metal oxides with (NH_4_)_2_SO_4_ at different temperatures.

### 3.2 Optimization of (NH_4_)_2_SO_4_ roasting parameters

Fig. 3(b)–(d) show the trends in the leaching efficiencies of valuable metals under different conditions. Fig. 3(b) shows the effect of (NH_4_)_2_SO_4_ dosage on the leaching efficiencies of Mg, Fe, and Ni at a fixed roasting temperature of 650 °C and a roasting time of 1.5 h. As the dosage increased from 0.5 to 2.5 g g^−1^, the leaching efficiencies of Mg and Ni increased significantly, reaching approximately 100% at 2.5 g g^−1^, respectively. In contrast, the leaching efficiency of Fe remained very low over the entire dosage range, with a maximum of only about 14%. This is because the roasting temperature of 650 °C exceeds 550 °C, above which Fe_2_(SO_4_)_3_ begins to decompose into insoluble iron oxides, thereby deteriorating the subsequent water leaching of Fe.^21,22^ Therefore, under these conditions, the Fe leaching efficiency is mainly controlled by the roasting temperature rather than the (NH_4_)_2_SO_4_ dosage. Based on the highest leaching efficiencies of Mg and Ni, 2.5 g g^−1^ was selected as the optimal (NH_4_)_2_SO_4_ dosage for further optimization. The excess dosage (2.5 g g^−1^) compared to the stoichiometric requirement (∼1.3 g g^−1^) compensates for the volatilization of the active sulfating gases at elevated temperatures and ensures sufficient molten phase for optimal solid–solid mass transfer.

**Fig. 3:**
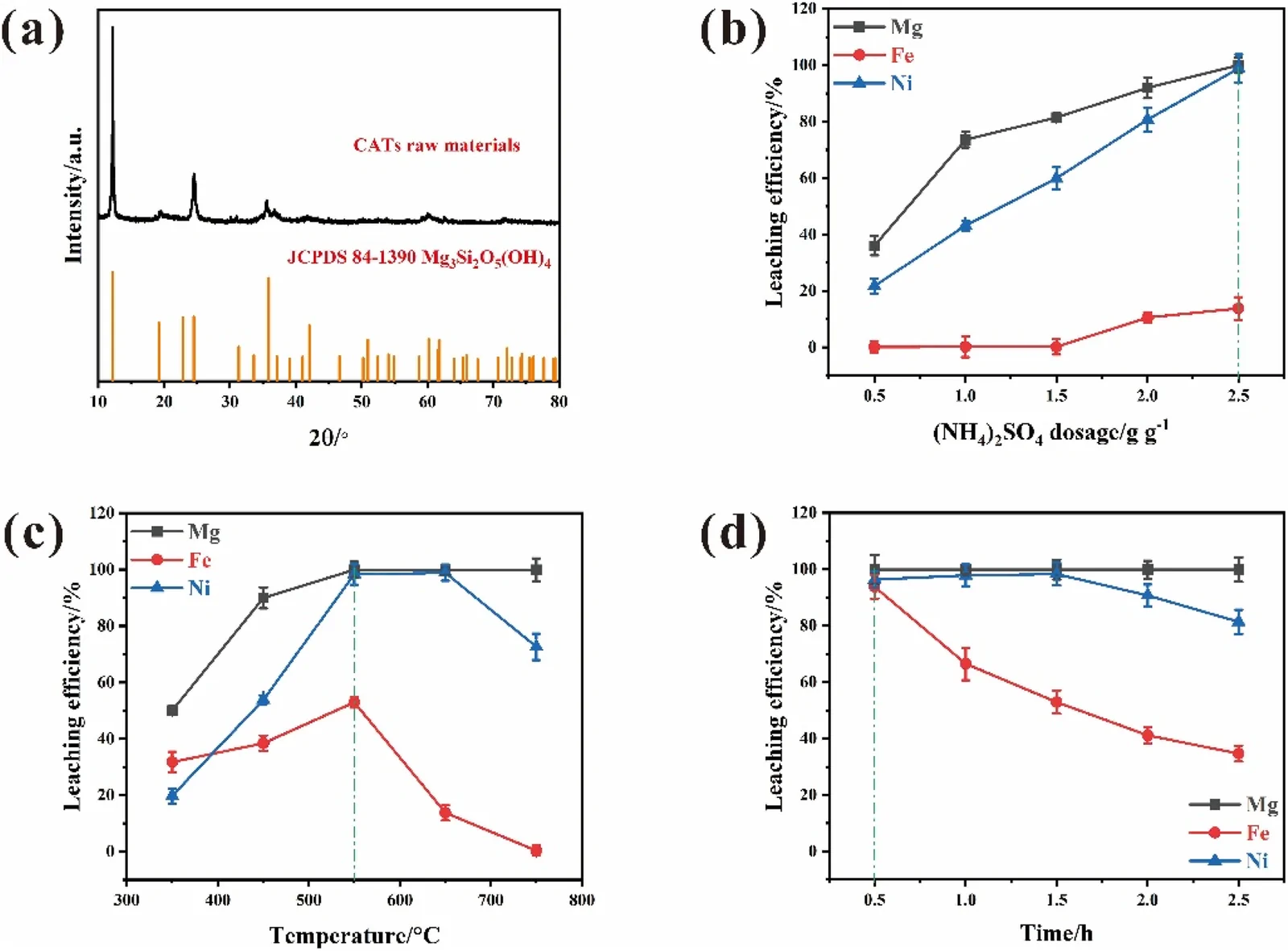
(a) XRD patterns of CATs powder. Effect of (b) (NH_4_)_2_SO_4_ dosage, (c) temperature and (d) time on leaching efficiencies of Mg, Fe and Ni.

Fig. 3(c) shows the effect of roasting temperature on the leaching efficiencies of Mg, Fe, and Ni with a fixed (NH_4_)_2_SO_4_ dosage of 2.5 g g^−1^ and a roasting time of 1.5 h. The leaching efficiencies of Mg and Ni increased significantly with rising temperature and reached high values at 550 °C. The Fe leaching efficiency also peaked at 550 °C. However, once the temperature exceeded 550 °C, the Fe leaching efficiency dropped rapidly, which is attributed to the decomposition of soluble Fe_2_(SO_4_)_3_ into insoluble iron oxides. When the temperature further exceeded 650 °C, the Ni leaching efficiency began to decline due to the decomposition of NiSO_4_ into nickel oxides.^23,24^ Therefore, 550 °C was selected as the optimal roasting temperature to ensure the simultaneous high leaching of Mg, Fe, and Ni. It should be noted that the 100 °C temperature step was designed to capture the macroscopic leaching trends for process optimization. The leaching efficiencies of Mg (99.9%) and Ni (98.34%) at 550 °C have already approached the theoretical limits, with the increase prior to 550 °C being driven by kinetic factors and enhanced lattice destruction. Therefore, a finer temperature grid (25–50 °C steps) would not yield higher extraction rates. Furthermore, the dosage study at 650 °C was specifically chosen to evaluate whether excess ammonium sulfate could suppress the thermal decomposition of Fe sulfates and ensure complete co-extraction.

Fig. 3(d) presents the effect of roasting time on the leaching efficiencies of Mg, Fe, and Ni under the fixed conditions of (NH_4_)_2_SO_4_ dosage of 2.5 g g^−1^ and roasting temperature of 550 °C. The leaching efficiencies of Mg and Ni reached high values within 0.5 h, and Mg remained almost stable thereafter. However, the Fe leaching efficiency began to decrease continuously after 0.5 h, probably because Fe_2_(SO_4_)_3_ gradually decomposed into iron oxides during prolonged roasting at 550 °C. When the roasting time exceeded 1.5 h, the Ni leaching efficiency also started to decline, which can be attributed to the conversion of NiSO_4_ into nickel oxide. Therefore, 0.5 h was selected as the optimal roasting time to ensure the simultaneous high leaching of Mg, Fe, and Ni while avoiding the decomposition of the soluble sulfates.

Through systematic optimization of (NH_4_)_2_SO_4_ dosage, roasting temperature, and roasting time, the optimal conditions for (NH_4_)_2_SO_4_ roasting of CATs were determined as follows: an (NH_4_)_2_SO_4_ dosage of 2.5 g g^−1^, a roasting temperature of 550 °C, and a roasting time of 0.5 h. A sufficient dosage ensured the complete sulfation of Mg, Fe, and Ni, while the moderate temperature of 550 °C avoided the decomposition of Fe_2_(SO_4_)_3_ into iron oxides that occurs above this temperature. Meanwhile, the short roasting time of 0.5 h prevented the gradual decomposition of ferric sulfate and NiSO_4_ into their respective oxides during prolonged heating. Under these optimized conditions, the leaching efficiencies of Mg, Fe, and Ni reached 99.9%, 93.8%, and 96.3%, respectively, achieving efficient co-extraction of multiple valuable metals and demonstrating the feasibility and superiority of the proposed process.

It should be noted that the current water-leaching step yields a dilute mixed leachate containing Mg, Fe, and Ni, which serves as an intermediate stream for metal recovery. To complete the flowsheet, a downstream separation process is proposed. Given the distinct precipitation behaviors of these metals, Fe can be initially removed *via* hydrolysis through precise pH adjustment to form Fe(OH)_3_/FeOOH precipitates. Subsequently, Ni and Mg can be separated from the purified solution using solvent extraction (*e.g.*, D2EHPA) or selective precipitation. This downstream separation will be the focus of our future work.

### 3.3 Thermal behavior and phase evolution

Following the determination of the optimal process parameters, the phase transformation and microstructural evolution during (NH_4_)_2_SO_4_ roasting were systematically investigated to elucidate the underlying reaction mechanism. The thermal behavior, phase composition, and morphology of the samples were characterized, and the reaction pathways were integrated into the discussion of each stage.

The thermal decomposition behavior of the CATs and (NH_4_)_2_SO_4_ mixture was investigated by TG-DTG analysis from room temperature to 800 °C, as shown in Fig. 4. Based on the TG curve and DTG peaks, the roasting process can be divided into three distinct stages. Stage 1 covers the temperature range from room temperature to approximately 350 °C. In the initial period from room temperature to 250 °C, the TG curve remains nearly flat with only a slight weight loss, corresponding to the evaporation of physically adsorbed water and a small amount of crystal water, and no significant chemical reaction occurs.^25^ As the temperature rises to 250–350 °C, a distinct weight loss begins, with a prominent DTG peak appearing at 313 °C. The total weight loss in this stage is approximately 14.55%, which is attributed to the decomposition of (NH_4_)_2_SO_4_ into NH_4_HSO_4_ and the release of NH_3_.^26,27^ The resulting active species subsequently reacts with the chrysotile structure, initiating the sulfation reaction and the initial breakdown of the Mg_3_Si_2_O_5_(OH)_4_ lattice. Stage 2 spans from 350 to 430 °C, where a distinct DTG peak appears at 407 °C. This stage exhibits a major weight loss of 35.36%, which is attributed to the extensive decomposition of the serpentine lattice and the rapid sulfation reaction. During this stage, the Mg_3_Si_2_O_5_(OH)_4_ structure breaks down substantially, and the metal-bearing phases are transformed into intermediate ammonium metal sulfates. This represents the most intense reaction period of the roasting process. Stage 3 extends from 430 to 800 °C, with a DTG peak at 447 °C. An additional weight loss of 13.21% occurs in this stage, during which the intermediate sulfates further react and convert into water-soluble sulfates, namely MgSO_4_, Fe_2_(SO_4_)_3_, and NiSO_4_. This corresponds to the optimal roasting temperature of 550 °C. However, when the temperature exceeds 600 °C, Fe_2_(SO_4_)_3_ begins to decompose into insoluble Fe_2_O_3_, and above 650 °C, NiSO_4_ gradually decomposes into NiO. These decomposition reactions account for the weight loss in the high-temperature region and directly explain the sharp decrease in Fe and Ni leaching efficiencies observed in the condition experiments at elevated temperatures. It should be noted that the distinct DTG peaks (*e.g.*, at 407 °C) primarily reflect the rapid decomposition and volatilization of the ammonium sulfate phase rather than the completion of metal sulfation. Although the thermodynamic driving force (Δ*G* < 0) for sulfation becomes favorable above 400 °C, a higher temperature (*e.g.*, 550 °C) is required to provide sufficient kinetic energy for the complete solid-state reaction and structural breakdown of serpentine.

**Fig. 4:**
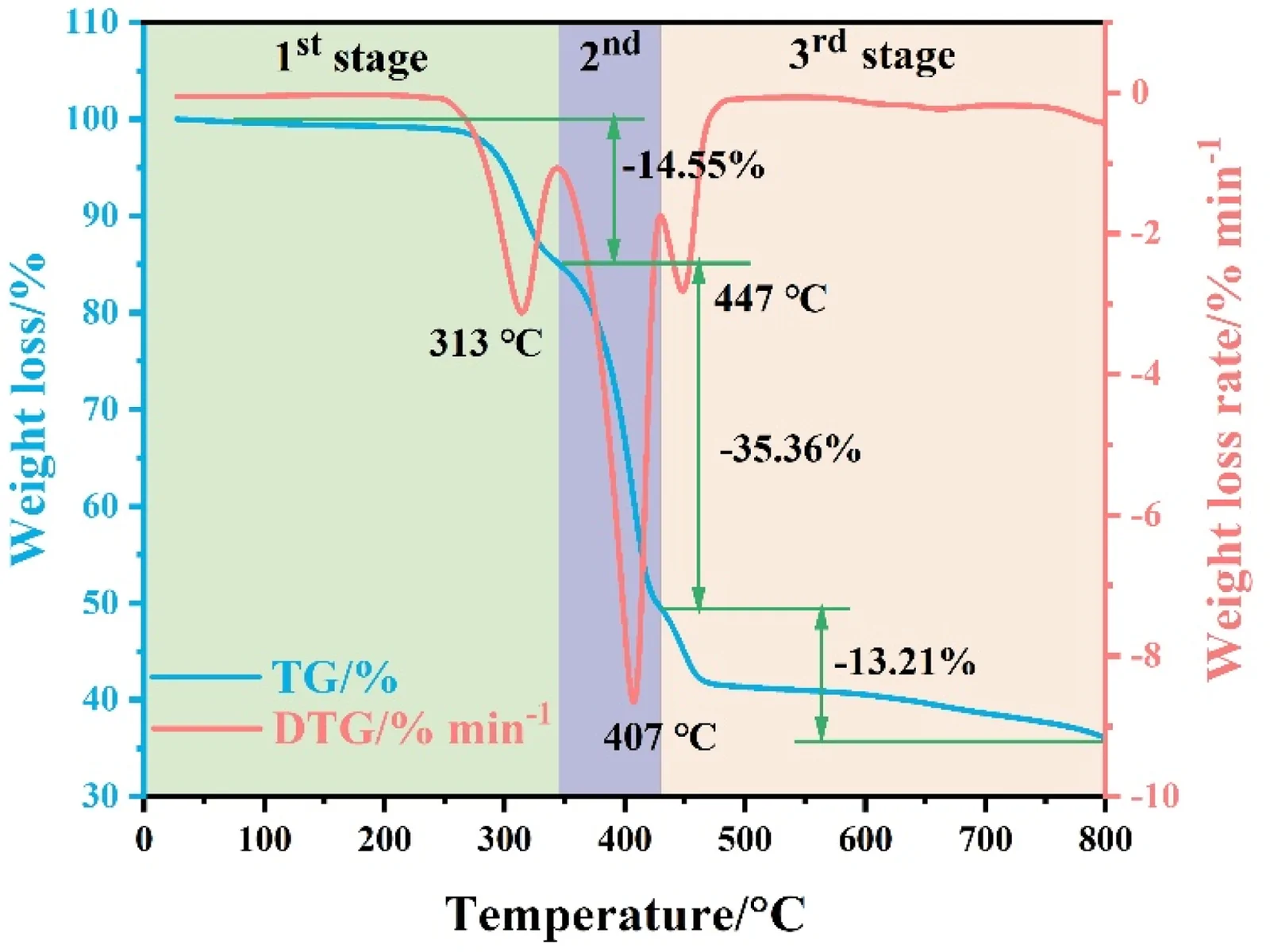
TG/DTG curves for the (NH_4_)_2_SO_4_ roasting process.

To further evaluate the reaction completeness and the appropriateness of the heating rate (10 °C min^−1^), a comparison between the theoretical and experimental weight loss was conducted. The theoretical weight loss for the complete sulfation reaction is calculated to be 60% (based on the stoichiometry of the main reactions). The experimental TG curve shows a total weight loss of 63.12% at 800 °C. The good agreement between the theoretical and experimental values indicates that the heating rate adopted in this study provides sufficient time for heat and mass transfer, ensuring the complete progress of the sulfation reaction without severe thermal lag.

In summary, the TG-DTG results confirm that 550 °C is the optimal temperature window, where the sulfation reaction is sufficiently complete, while the decomposition of Fe_2_(SO_4_)_3_ and NiSO_4_ is effectively avoided.

Fig. 5(a) presents the XRD patterns of the roasted products obtained at 350–750 °C. At 350 °C, the characteristic peaks A, B, and C assigned to the CATs structure remain visible, indicating that the serpentine lattice is still largely retained. As the temperature increases to 450 and 550 °C, the intensities of these peaks decrease markedly, while the diffraction peaks of MgSO_4_, Fe_2_(SO_4_)_3_, and NiSO_4_ appear and become dominant at 550 °C, suggesting that the sulfation reaction proceeds extensively. When the temperature further rises to 650 and 750 °C, the peaks of Fe_2_(SO_4_)_3_ and NiSO_4_ gradually weaken, indicating their decomposition at elevated temperatures. This decomposition explains the sharp decrease in Fe and Ni leaching efficiencies above 600 °C and 650 °C, respectively.

**Fig. 5:**
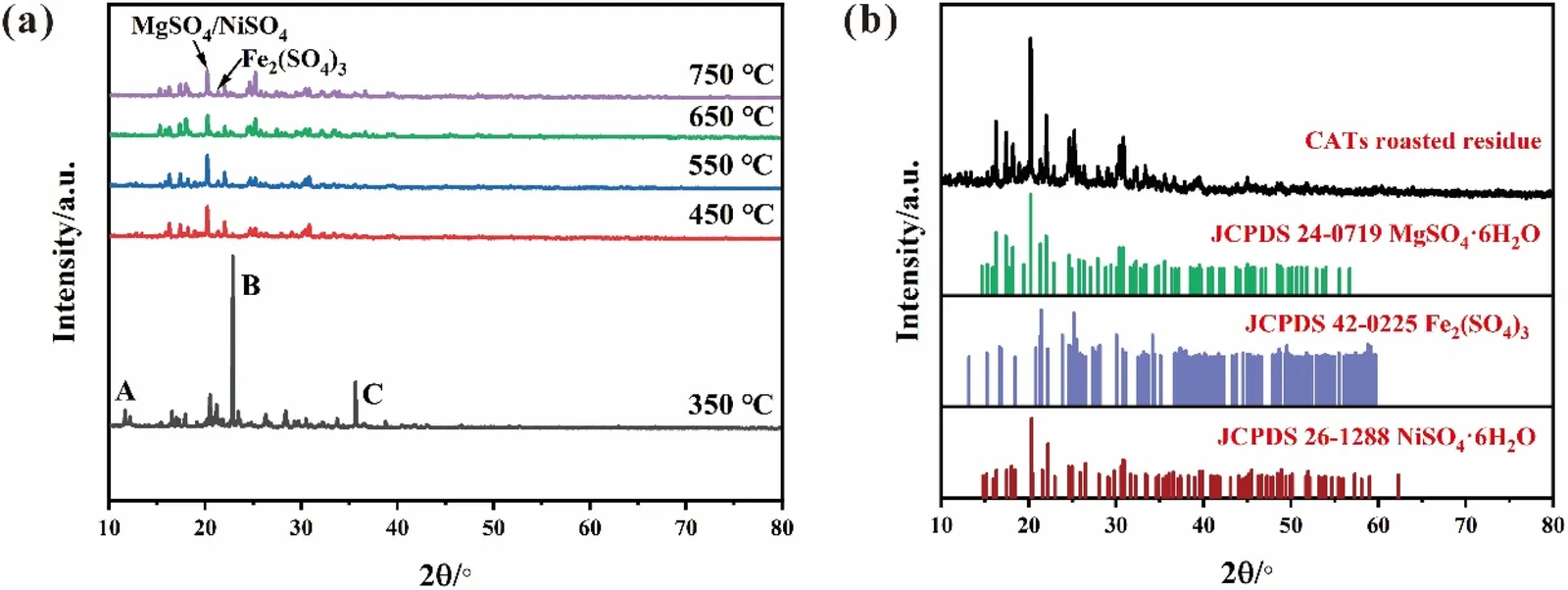
XRD patterns of roasted residue (a) at different temperatures and (b) under optimal conditions.

Fig. 5(b) shows the XRD pattern of the roasted product obtained under the optimal conditions (2.5 g per g (NH_4_)_2_SO_4_, 550 °C, 0.5 h). The diffraction peaks match well with the standard patterns of MgSO_4_·6H_2_O (JCPDS 24-0719), Fe_2_(SO_4_)_3_ (JCPDS 42-0225), and NiSO_4_·6H_2_O (JCPDS 26-1288). No significant peaks of unreacted chrysotile or insoluble iron/nickel oxides are detected, indicating that Mg, Fe, and Ni in the CATs have been effectively transformed into water-soluble sulfates. The absence of Fe_2_O_3_ and NiO peaks confirms that the decomposition of ferric sulfate and nickel sulfate is successfully avoided at 550 °C. Meanwhile, the silicon component remains in the residue, likely in the form of amorphous SiO_2_, which is consistent with the absence of sharp SiO_2_ diffraction peaks. These results provide direct mineralogical evidence for the high co-extraction efficiencies of Mg (99.9%), Fe (93.8%), and Ni (96.3%) under the optimal conditions.

### 3.4 Microstructural and elemental evolution

Fig. 6 shows the SEM images and EDS elemental mapping of the roasted product obtained under the optimal conditions. The low-magnification SEM image reveals that the roasted product consists of irregular agglomerates with a rough and loose surface, in contrast to the dense and fibrous morphology of the raw CATs. The high-magnification image further shows that the particle surface is porous and exhibits layered or flaky structures, which is a typical feature of structural collapse and volume expansion caused by the decomposition of the serpentine lattice. This morphological change provides direct evidence that the (NH_4_)_2_SO_4_ roasting process effectively destroys the original chrysotile structure, exposing the internal metal-bearing phases to the sulfation reaction.

**Fig. 6:**
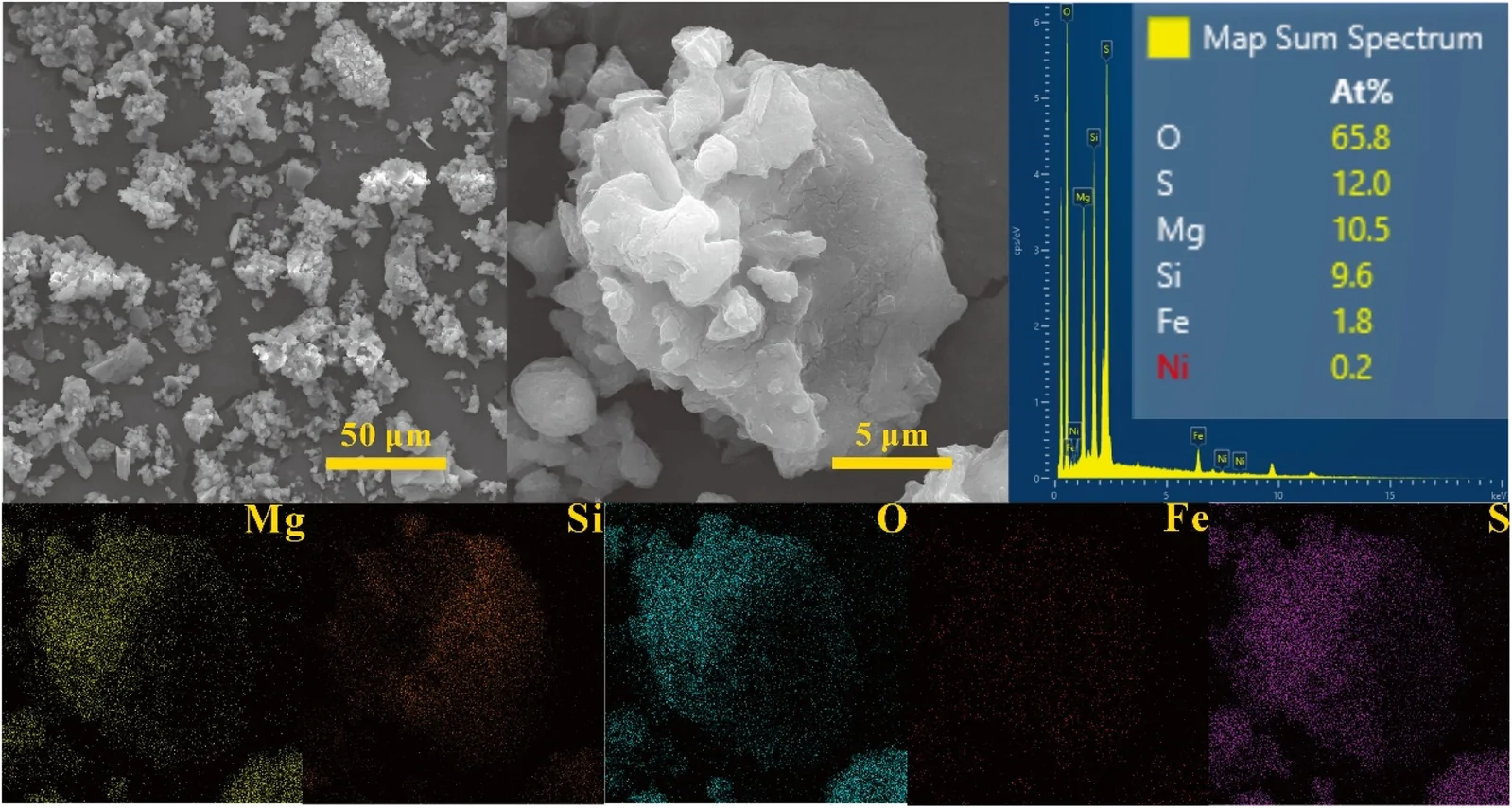
SEM images and EDS-mapping results of CATs roasted powder.

The EDS mapping results demonstrate that Mg, Fe, and S are homogeneously distributed and highly overlapped within the roasted particles, confirming that the metallic elements have been transformed into sulfate compounds. The EDS spectrum shows that the atomic percentages of O, S, Mg, Si, Fe, and Ni are 65.8%, 12.0%, 10.5%, 9.6%, 1.8%, and 0.2%, respectively. The significant presence of S (12.0 at%) strongly supports the formation of MgSO_4_, Fe_2_(SO_4_)_3_, and NiSO_4_. Meanwhile, Si remains uniformly distributed throughout the residue without overlapping with S, indicating that Si is not sulfated and remains in the form of amorphous SiO_2_. This confirms that the sulfation reaction selectively converts the metal components into water-soluble sulfates while leaving the silicon framework intact, which is consistent with the XRD and leaching results.

### 3.5 Morphological evolution and fiber destruction

The morphological evolution of the raw serpentine and the final water-leaching residue was further examined by TEM, and the result is shown in Fig. S1. The raw serpentine exhibits abundant slender fibrous structures, whereas such fibrous structures are almost absent in the water-leaching residue, which is mainly composed of irregular granular and porous aggregates. This contrast indicates that the fibrous framework of serpentine was largely destroyed during ammonium sulfate roasting-water leaching, which is consistent with the dehydroxylation, structural breakdown and sulfation of Mg, Fe and Ni revealed by XRD and leaching results.

### 3.6 Proposed mechanism of (NH_4_)_2_SO_4_-assisted roasting

During heating, ammonium sulfate decomposes to release NH_3_ and generate active sulfate species such as NH_4_HSO_4_, (NH_4_)_2_S_2_O_7_ and SO_3_, which attack the Mg–O bonds and the Fe/Ni-bearing sites in serpentine, promoting dehydroxylation and breakdown of the serpentine framework. As a result, Mg, Fe and Ni are converted into water-soluble sulfates, while Si is mainly retained as insoluble SiO_2_. The release of NH_3_ and H_2_O also creates a porous roasted product, which improves mass transfer during subsequent water leaching and enables efficient co-extraction of Mg, Fe and Ni rather than selective extraction of Mg alone.

### 3.7 Environmental implications

The (NH_4_)_2_SO_4_ roasting-water leaching process provides a green and sustainable pathway for the utilization of hazardous CATs. Compared with conventional acid leaching, this process avoids the use of strong acids and thus eliminates serious equipment corrosion and the generation of large volumes of acidic wastewater. (NH_4_)_2_SO_4_ acts as a recyclable sulfation agent, and water is employed as the sole leaching medium, making the whole process chemically benign. The NH_3_ and SO_*x*_ gases released during roasting can be captured and returned to the process, effectively reducing harmful gaseous emissions. In addition, the relatively low roasting temperature and short reaction time significantly lower energy consumption. The final leaching residue is composed mainly of amorphous silica, which is environmentally inert and can be further used as a potential silicon resource, thereby minimizing the amount of secondary solid waste. Overall, this process achieves efficient co-extraction of Mg, Fe, and Ni from hazardous tailings while reducing chemical consumption, energy input, and environmental risks, demonstrating a cleaner production strategy for the sustainable valorization of industrial solid waste.

## 4. Conclusion

An (NH_4_)_2_SO_4_ roasting-water leaching process was developed for the co-extraction of Mg, Fe, and Ni from CATs. The optimal conditions were an (NH_4_)_2_SO_4_ dosage of 2.5 g g^−1^, a roasting temperature of 550 °C, and a roasting time of 0.5 h. Under these conditions, the leaching efficiencies of Mg, Fe, and Ni reached 99.9%, 93.8%, and 96.3%, respectively. TG-DTG, XRD, and SEM-EDS analyses revealed that the serpentine structure was destroyed and the metals were converted into water-soluble MgSO_4_, Fe_2_(SO_4_)_3_, and NiSO_4_, while Si remained as amorphous SiO_2_. Higher temperatures or longer roasting times caused the decomposition of Fe_2_(SO_4_)_3_ and NiSO_4_ into insoluble oxides, reducing Fe and Ni leaching. This process avoids the use of strong acids and employs water as the leaching medium, offering a cleaner route for the sustainable utilization of hazardous CATs.

## Conflicts of interest

The authors declare no competing financial interest.

## Supplementary Material

RA-OLF-D6RA09542A-s001

## Data Availability

The data supporting this article are included within the article. Additional data are available from the corresponding author on reasonable request. Supplementary information (SI) is available. See DOI: https://doi.org/10.1039/d6ra09542a.
