# Supplementary material for: Extraction of critical metals from chrysotile asbestos tailings via an ammonium sulfate roasting-water leaching process
Source: RSC Adv. 2026 Nov 12. Online ahead of print. doi: 10.1039/d6ra09542a (PMC13757420; doi:10.1039/d6ra09542a)
Supplement: Supplementary file 1 [file RA-OLF-D6RA09542A-s001.pdf]

Supplementary Information (SI) for RSC Advances.
This journal is © The Royal Society of Chemistry

Extraction of Critical Metals from Chrysotile Asbestos Tailings via Ammonium Sulfate Roasting-Water Leaching Process

Hongxiang Gan[a], Xiangyang Zhou[a], Fanyun Su[a, *]

[a] *School of Metallurgy and Environment, Engineering Research Center of the Ministry of Education for Advanced Battery Materials, Central South University, Changsha 410083, China*

*Corresponding Author: Fanyun Su. Tel. 13278881878. E-mail Address: 1453636044@qq.com.

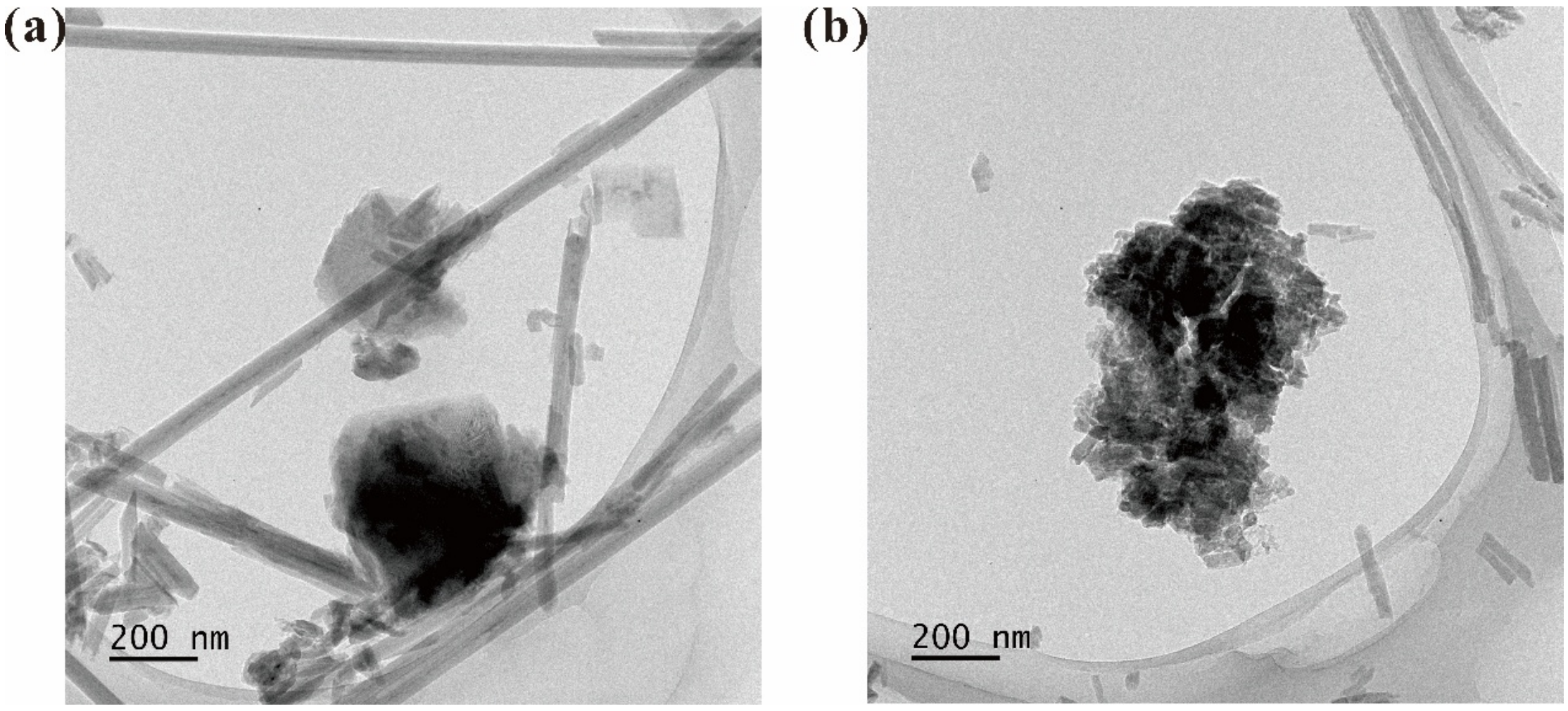

Fig.S1. TEM results of (a) the raw material CATs and (b) the water leaching residue.
